# Deciphering Protein Dynamics of the Siderophore Pyoverdine Pathway in *Pseudomonas aeruginosa*

**DOI:** 10.1371/journal.pone.0079111

**Published:** 2013-10-30

**Authors:** Laurent Guillon, Stephan Altenburger, Peter L. Graumann, Isabelle J. Schalk

**Affiliations:** 1 UMR 7242, Université de Strasbourg-CNRS, Strasbourg, France; 2 SYMMIKRO, LOEWE Center for Synthetic Microbiology, and Department of Chemistry, University of Marburg, Marburg, Germany; Ben-Gurion University of the Negev, Israel

## Abstract

*Pseudomonas aeruginosa* produces the siderophore, pyoverdine (PVD), to obtain iron. Siderophore pathways involve complex mechanisms, and the machineries responsible for biosynthesis, secretion and uptake of the ferri-siderophore span both membranes of Gram-negative bacteria. Most proteins involved in the PVD pathway have been identified and characterized but the way the system functions as a whole remains unknown. By generating strains expressing fluorescent fusion proteins, we show that most of the proteins are homogeneously distributed throughout the bacterial cell. We also studied the dynamics of these proteins using fluorescence recovery after photobleaching (FRAP). This led to the first diffusion coefficients ever determined in *P. aeruginosa*. Cytoplasmic and periplamic diffusion appeared to be slower than in *Escherichia coli* but membrane proteins seemed to behave similarly in the two species. The diffusion of cytoplasmic and periplasmic tagged proteins involved in the PVD pathway was dependent on the interaction network to which they belong. Importantly, the TonB protein, motor of the PVD-Fe uptake process, was mostly immobile but its mobility increased substantially in the presence of PVD-Fe.

## Introduction

Iron is an essential element for bacterial growth. Ions of this metal are involved in fundamental biological processes, including the respiratory chain, metabolic transformations and deoxyribonucleotide biosynthesis. To obtain the poorly soluble ions of this metal, bacteria produce small organic chelators called siderophores [1].

**Figure 1:**
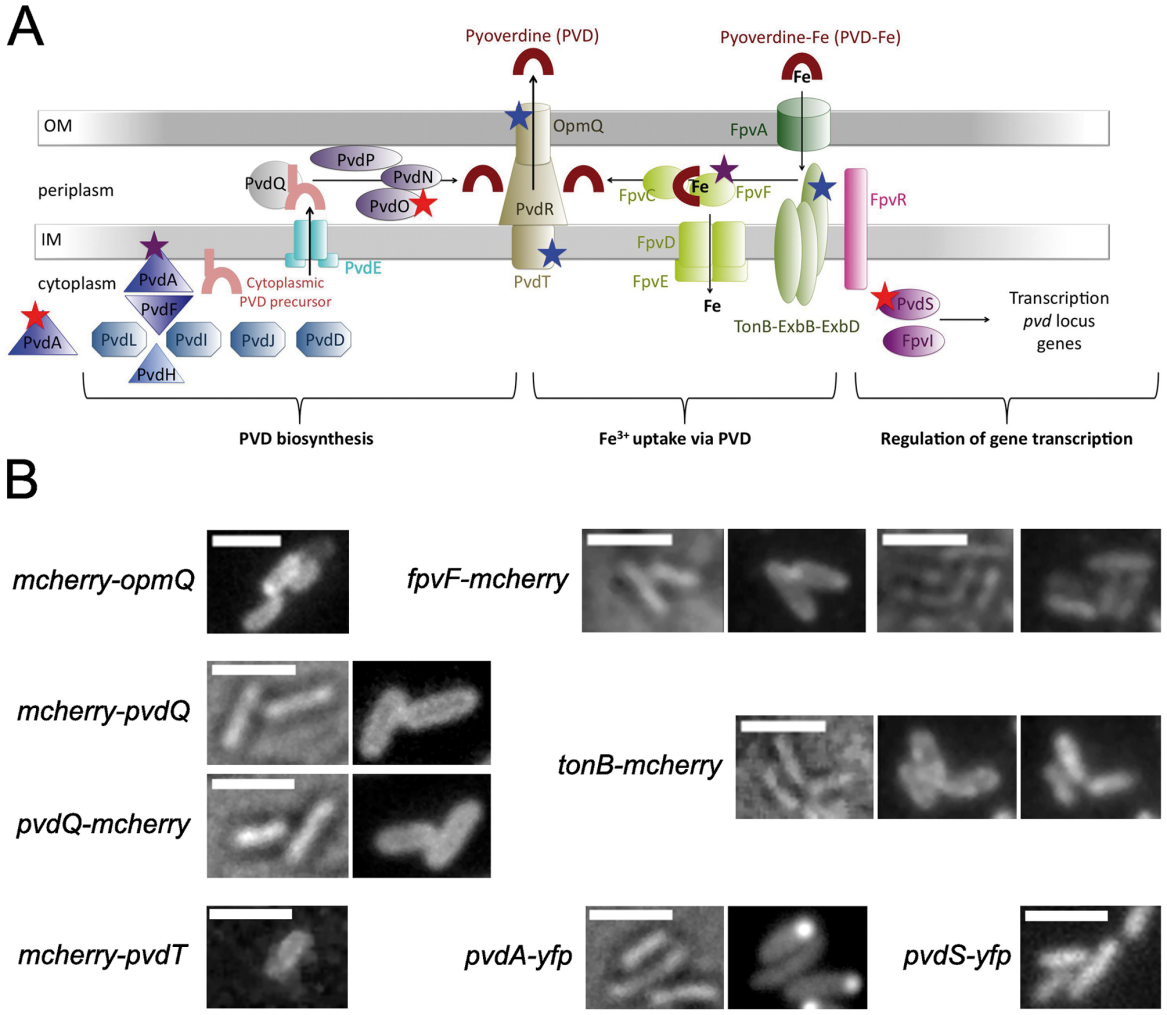
A. Scheme depicting the PVD pathway involving biosynthesis, iron uptake and gene expression. For details and explanations, refer to the “Introduction” section. The results obtained in this work on protein dynamics are indicated as follows: the stars in red, purple and blue indicate the proteins with rapid, moderate and slow dynamics, respectively. **B.** **Fluorescence microscopy analysis of fluorescent fusions with, from left to right and up to down, OpmQ, FpvF, PvdQ, TonB, PvdT, PvdA and PvdS.** Cells were grown twice in minimal medium, washed in minimal medium and spotted onto slides coated with agarose made up in minimal medium. Brightfield images, when available, are presented on the left. Due to low fluorescent signals, epifluorescence images of *pvdS-yfp*, *mcherry-pvdT* and *mcherry-opmQ* were recorded using a high sensitivity camera. Images of *fpvF-mcherry* in both epifluorescence (left panel) and TIRF (right panel) are shown. Epifluorescence images of *pvdA-yfp*, *mcherry-pvdQ* and *pvdQ-mcherry* are presented. For *tonB-mcherry,* from left to right, brightfield, epifluorescence and TIRF mode images are presented (scale bar 2 µm). For the fluorescence miscrocopy pictures of PAO1 strain harboring a plasmid encoding a cytoplasmic mCHERRY fluorescent protein expressed under the control of the *pvdA* promoter (PAO1(pMMB-*mcherry*)) see in Supplemental Materials (Figure 5-SM).

The opportunistic pathogen *Pseudomonas aeruginosa* synthesizes a major fluorescent siderophore, pyoverdine (PVD) [2], [3]. Like all siderophore pathways, the processes from PVD biogenesis to PVD-dependent iron uptake are tightly regulated by complex mechanisms, involving machineries that span bacterial membranes [2], [3] (**Figure 1A**). PVD biosynthesis starts in the cytoplasm by the action of four non-ribosomal peptide synthetases (NRPSs), PvdL, PvdI, PvdJ and PvdD [3], [4], which produce a precursor peptide acylated with a myristic or myristoleic chain at the beginning of the process [3], [5]. This peptide contains unusual amino acids that are provided from the cytoplasmic enzymes PvdA, PvdF and PvdH [3], [4]. The presence of a fatty chain may retain the PVD precursor at the inner membrane during its assembly [5], consistent with our recent finding that PvdA interacts with the inner membrane [6]. The cytoplasmic precursor probably crosses the inner membrane through the PvdE ABC-transporter [7]. Once in the periplasm, the molecule is matured further into the fluorescent PVD by PvdN, PvdO, PvdP and PvdQ enzymes [5], [7]–[10]. PvdQ, an N-terminal nucleophile hydrolase (Ntn-hydrolase), removes the fatty chain [5], [11] and PvdN, PvdO and PvdP appear to be involved in chromophore cyclization [7]. Newly synthesized PVD is secreted into the extracellular medium from the periplasm by the efflux pump PvdRT-OpmQ [12].

Following chelation of iron in the extracellular medium, the PVD-Fe complex is recognized by FpvA, its specific outer membrane transporter [13]. FpvA is composed of three domains: a C-terminal β-barrel inserted in the outer membrane; a plug domain that occupies the lumen of the β-barrel; and a N-terminal periplasmic domain called the signaling domain [14], [15]. Residues of both plug and β-barrel domains on the extracellular face of the transporter are involved in ferric siderophore recognition [15], [16]. Subsequent internalization of the ferrisiderophore into the periplasm through FpvA requires energy, provided by the TonB-ExbB-ExbD complex, but the molecular mechanism involved remains unknown [17]. A region of four to five residues between the signaling and the plug domains of FpvA, called the TonB box, is involved in the transporter energization [18]. This region allows the interaction between the TonB protein and the outer membrane transporter [18]–[20] and therefore the energy transfer from TonB to FpvA. The mechanism of channel formation leading to import of the substrate through the outer membrane also remains to be elucidated but simulations indicate the interaction between TonB and the TonB box would lead to the plug domain unfolding [21]. In the genome of *P. aeruginosa*, three *tonB* genes have been identified but only *tonB1* was found to be involved in PVD-dependent iron uptake [22]. Following its import into the periplasm, the PVD-Fe complex is dissociated through an iron reduction process [23]. The enzymes involved in this process are yet to be identified but the recent identification of an ABC-transporter FpvCDEF may give the first leads [24]. This transporter possesses the unusual characteristics of having two periplasmic binding proteins (PBPs): FpvC and FpvF. These two proteins form complexes that bind ferric pyoverdine in the bacterial periplasm [24] before iron release from the siderophore. Once dissociated from iron, the apo-PVD is recycled into the extracellular media by the efflux pump PvdRT-OpmQ [25]–[27].

To maintain iron homeostasis, the whole PVD pathway is tightly regulated by the master ferric uptake repressor, Fur, and PVD-specific regulation [28]. The PVD-specific regulation involves the N-terminal signaling domain of FpvA and an anti-sigma factor, FpvR, and its two cognate alternative sigma factors, PvdS and FpvI [29]. FpvI promotes the transcription of the *fpvA* gene coding for the outer membrane transporter and PvdS activates the transcription of all other genes of the pathway [30]. The inner membrane FpvR binds both PvdS and FpvI, preventing them from activating the transcription in the cytoplasm [31]. Binding of PVD-Fe to FpvA induces a conformational change leading to signaling domain exposure, presumably facilitating interaction with FpvR [14], [32]–[35]. This may then trigger the release of the two sigma factors such that would they can act on transcription.

Fusions to fluorescent proteins are powerful tools to obtain insights on the protein distribution in bacterial cells. We recently described this type of approach for investigating two enzymes involved in PVD biosynthesis: PvdA and PvdQ [6]. We showed that the cytoplasmic PvdA was mostly present at the old cell pole whereas PvdQ, involved in PVD maturation, was uniformly distributed throughout the periplasm. In addition to such cellular localization information, fluorescently tagged proteins can provide valuable insights into dynamic processes. Indeed, fluorescence recovery after photobleaching (FRAP) is a powerful technique for studying the dynamics of a labeled protein, a critical parameter for its function. This technique has been widely used in the model organism *Escherichia coli* and led to better characterization of various individual proteins, including cytochrome *bd*-I [36] and outer membrane proteins [37]–[39], and small multi-component systems, such as the chemotaxis system [40], [41].

We extended our previous strategy of fluorescence tagging of proteins involved in PVD biosynthesis to proteins participating in other steps of the pathway. We believe that this is the first time a whole siderophore pathway has been visualized. We show that proteins involved in PVD efflux, uptake or regulation were homogeneously distributed throughout the cell compartment they belong to. In addition to describing the distribution of the proteins of the pathway, we also used the FRAP technique to study their dynamics. This pioneering study in *P. aeruginosa* provides a basis for comparing the dynamics between this organism and *E. coli*. We found that the main rules governing diffusion in the three cell compartments of *E. coli* are transposable to *P. aeruginosa*. Diffusion in both the cytoplasm and the periplasm, however, was slower in *P. aeruginosa*, whereas the speed of diffusion of membrane proteins was similar in the two species. Last but not least, we showed that a substantial fraction (90%) of TonB, the central player for iron uptake, was immobile but that its mobility is increased up to 35% upon activation of the system.

## Materials and Methods

### Bacterial strains, plasmids, and growth conditions

The *P. aeruginosa* strains and the *E. coli* strains used in this study are listed in Table 1 and the plasmids in Table S1 of File S1. Bacteria were routinely grown in LB broth (Difco) medium at 37°C. *P. aeruginosa* strains were grown overnight at 30°C in an iron-deficient succinate medium (composition: 6 g/L K_2_HPO_4_, 3 g/L KH_2_PO_4_, 1 g/L (NH_4_)_2_SO_4_, 0.2 g/L MgSO_4_.7H_2_O, and 4 g/L sodium succinate, with the pH adjusted to 7.0 by adding NaOH). Gentamycin (50 µg mL^−1^) was added when required. The strategy used to construct the mutants used in this study is described in File S1 of Supporting Information as well as the list of oligonucleotides and plasmids used in this study (**Table S1** and **S2** in File S1).

**Table 1:** Strains used in this study.

Strain or plasmid	CollectionID	Relevant characteristics	Source or reference
*P. aeruginosa* strains			
PAO1		wild-type strain	[63]
PAO1*tonB*	PAD08	derivative of PAO1; Δ*tonB*::Tc	[49]
PAO1*fpvC*		derivative of PAO1; Δ*fpvC*::WGenta	[9]
PAO1*fpvCDEF*		derivative of PAO1; Δ*fpvCDEF,*	[24]
PAO1*pvdRTopmQ*		derivative of PAO1; Δ*pvdRTopmQ,*	[8]
PAO1*pvdS*	PAS161	derivative of PAO1; Δ*pvdS*,	this study
*pvdA-yfp*	PAS102	derivative of PAO1; *pvdA-eyfp*,	[6]
*mcherry-pvdQ*	PAS065	derivative of PAO1; *pvdQ-mcherry*, mCHERRY insertion after A23 of PvdQ,	[6]
*pvdQ-mcherry*	PAS069	derivative of PAO1; *pvdQ-mcherry*,	[6]
*pvdS-yfp*	PAS120	derivative of PAO1; *pvdS-yfp*, chromosomally integrated	this study
*fpvF-mcherry*	PAS109	derivative of PAO1; *fpvF-mcherry*,	this study
*tonB-mcherry*	PAS083	derivative of PAO1; *tonB-mcherry*,	this study
*mcherry-pvdR*	PAS127	derivative of PAO1; *mcherry-pvdR*, mCHERRY insertion after A25 of PvdR,	this study
*mcherry-pvdT*	PAS129	derivative of PAO1; *mcherry-pvdT*,	this study
*mcherry-opmQ*	PAS137	derivative of PAO1; *mcherry-opmQ*, mCHERRY insertion after A19 of OpmQ, chromosomally integrated	this study
*E. coli* strains			
TOP10		*supE44 DlacU169 (φ80lacZDM15) hsdR17 recA1 endA1 gyrA96 thi-1relA1*	Invitrogen
S17-1		*pro thi hsdR recA*; chromosomal RP4 (Tra+ Tcs Kms Aps); Tpr Smr	[64]

The sequences encoding all fluorescent tags were integrated into the chromosome.

### Phenotypic characterization of fluorescently labeled strains

The methodology used for characterizing the mutant strains by studying their growth and PVD production, and by immunoblot analysis, cellular fractionation, iron uptake assays and assaying periplasmic PVD is described in the corresponding sections of the Supplemental Materials.

### Fluorescence and FRAP microscopy imaging

Samples were prepared from strains cultured overnight in iron-depleted minimal medium. Cultures were washed in minimal medium and appropriately diluted in the same medium; 5 µL of the cell suspension was spotted on a glass slide that was freshly coated with 1% agarose in minimal medium, and covered with a cover slip.

Epifluorescence images of strains *pvdA-yfp, mcherry-pvdQ* and *pvdQ-mcherry* were acquired with a Nikon 50i (objective: CFI Achroplan 100× A ON 1.25 DT 0.18) microscope equipped with a numeric 12 bits DS-Fi1 camera. For fluorescence imaging, GFP-3035B (excitation 472±32 nm, emission 520±35 nm, dichroic filter 502–730 nm) and TRITC HYQ (excitation 545±15 nm, emission 620±30 nm, dichroic filter 570 longpass) filters were used for imaging eYFP and mCHERRY, respectively. Images were captured using imaging software NIS elements.

Epifluorescence images of strains *mcherry-pvdT, mcherry-opmQ* and *pvdS-yfp* and FRAP streams were acquired on an Observer.Z1 (Zeiss) equipped with a Plan Fluar objective (NA: 1.45, Zeiss) and an Evolve 512 EMCCD camera (photometrics). Data was analyzed with Metamorph 7.7.3.0 software (Molecular Devices Inc.). Fluorophores were excited by exposure to a laser at a wavelength of 488 nm, 561 nm or 445 nm. Fluorescence signals were acquired using the appropriate filter cubes.

Epifluorescence and TIRF images were minimally processed with Image-J_NIH_ software [42].

### FRAP data analysis

FRAP Analysis plugin in Image-J_NIH_ software [42] was used for analysis of recovery. The fluorescence recovery was converted into a one-dimensional diffusion model to determine diffusion coefficients, as described previously [43]. Cell streams were rotated to align the long cell axis in the vertical dimension. The cell was sliced along this axis, removing the poles of the bacteria to avoid border problems. The intensities were summed in each slice to obtain one-dimensional intensity profiles 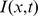 (the intensity I at position x and time t) using Image-J_NIH_ software [42]. The one-dimensional intensity profiles were used to calculate the Fourier amplitudes terms 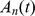:
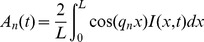



The Fourier terms for 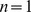 were considered because the higher 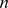-terms decay too quickly to be followed by video rate cameras. The 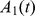 function was fitted to a single exponential decay, 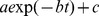, with 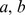 and 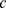 as free parameters. The diffusion constant was obtained from the decay rate, 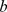, by the relation 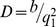. A macro developed in-house was used for data processing and fitting by non-linear regression using the Scilab environment (Scilab Enterprises. Scilab : open source, free, software for numerical calculations, (Mac OS X, Version 5.4) [software] (2012). available at http ://www.scilab.org). Statistical analysis of the data was performed in R environment [44].

## Results

### All the labeled proteins of the PVD pathway, except PvdA, are uniformly distributed throughout the bacteria cell compartment where they are expressed

We used the strategy we developed previously to insert DNA encoding fluorescent protein into the pseudomonas genome [6] such that constructs encoding translational fusions of the reporter to proteins involved in the PVD pathway were obtained. For each protein, both N- and C-terminal labelings were investigated. Bacterial growth and PVD production were monitored for each construction at 600 nm and 400 nm, respectively. Production of the fusion protein was checked by immunoblot analysis on whole cells. Here, we report and describe only fusion proteins with wild-type phenotypes for PVD production and secretion, and for iron uptake: PvdS-YFP (C-terminally labeled), FpvF-mCHERRY (C-terminally labeled), TonB-mCHERRY (C-terminally labeled), mCHERRY-PvdR (N-terminally labeled) and mCHERRY-PvdT (N-terminally labeled) (**Table 1**). We also present findings for mCHERRY-OpmQ (N-terminal fusion protein). The phenotypic characterization of the strains expressing all these fusion proteins are reported in the Supplemental materials (**Figures S1 to S4 in File S1**). Attempts to label FpvA, FpvR and FpvC were unsuccessful: either the fusion proteins were unstable, or PVD production and secretion or iron transport were affected.

**Figure 1B** shows the epifluorescence microscopy pictures of strains *pvdS-yfp, tonB-mcherry*, *fpvF-mcherry*, *mcherry-pvdR*, *mcherry-pvdT* and *mcherry-opmQ* (**Table 1**). Epifluorescence microscopy imaging shows PvdA (a cytoplasmic enzyme involved in PVD biosynthesis) clustered at the old cell pole of *P. aeruginosa* and PvdQ (a periplasmic enzyme involved in PVD biosynthesis) uniformly distributed all over the periplasm [6] and these images are provided here for reference (**Figures 1B** **)**. The fluorescent signal of the eYFP fusion with PvdS was weak, such that a high sensitivity camera was required: this fusion was uniformly distributed in the cytoplasmic compartment (**Figure 1B**). Epifluorescence and TIRF mode microscopy were used for all other strains. The mCHERRY fluorescence of *tonB-mcherry* (TonB being the activator of the outer membrane FpvA transporter) showed a homogeneous peripheral distribution indicating that the fusion proteins were uniformly distributed throughout the inner membrane (**Figure 1B**). This distribution is similar to that of GFP-TonB in *E. coli* [45]. For *fpvF-mcherry* (FpvF is involved in PVD-Fe uptake), the signal appeared as a fluorescence halo in epifluorescence, consistent with its periplasmic localization; the fusion protein was uniformly distributed in this cell compartment as evidenced by the TIRF imaging (**Figure 1B**).

The fluorescence signals for *mcherry-pvdR*, *mcherry-pvdT* and *mcherry-opmQ*, following cell fractionation, were associated with the membrane fraction; these strains retained wild-type efflux activity, except *mcherry-opmQ,* which accumulated PVD in the periplasm like a *pvdRTopmQ* deletion mutant (**Figure S4C in File S1**). Deletion of the PvdRT-OpmQ efflux pump leads to increased periplasmic accumulation of PVD [27], [46]. The periplasmic concentration of PVD in both *mcherry-pvdR* and *mcherry-pvdT* was similar to that in strain PAO1, whereas *mcherry-opmQ* accumulated more PVD than PAO1*pvdRTopmQ* (**Figure S4D in File S1**). Imaging of strain *mcherry-pvdR* did not yield a sufficient signal to localize the fusion. Both *mcherry-pvdT* (**Figure 1B**) and *mcherry-opmQ* (**Figure 1B**) were found by a high sensitivity camera in epifluorescence mode to have a uniform distribution throughout the membrane.

### PvdA, involved in PVD biosynthesis, and the sigma factor PvdS diffuse freely in the cytoplasm

**Figure 2:**
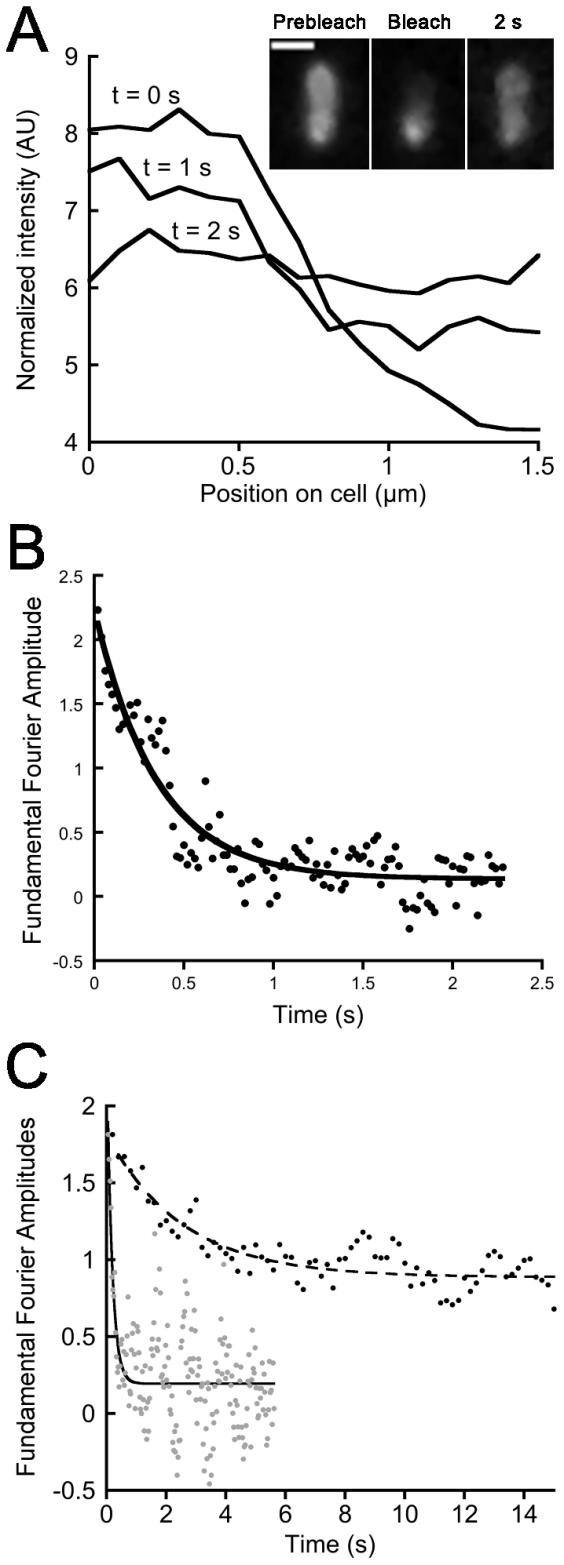
Illustration of FRAP data treatment for PvdQ-mCHERRY and comparison of the fast diffusing of PvdS-YFP with the slow diffusing TonB-mCHERRY. A. One-dimensional profiles along the long cell axis after photobleaching (t = 0 s), during recovery (t = 1 s) and after full recovery (t = 2 s) of strain *pvdQ-mcherry*. Inset: Fluorescence images extracted from the acquired FRAP stream. From left to right: before photobleaching, after photobleaching and after recovery. B. Experimental Fourier amplitudes for mode n = 1 as a function of time of PvdQ-mCHERRY and the fitted exponential decay (solid line) used to determine the diffusion coefficient. C. Experimental Fourier amplitudes for mode n = 1 as a function of time for PvdS-YFP (gray circles) and TonB-mCHERRY (black circles) and the corresponding fitted exponential decay (hashed and solid lines, respectively).

FRAP data were treated as described in [43]. Briefly, diffusion was reduced to a one dimension issue by slicing the bacteria along the long cell axis. The diffusion coefficient (D) was then evaluated from the decay rate of the amplitudes of the first Fourier mode, as illustrated in **Figure 2**. For all strains, no recovery could be detected after a whole cell had been bleached. This confirmed that the recovery we monitored arises only from diffusion of fluorescent molecules already present in the bacteria and not from *de novo* biosynthesis of proteins. The diffusion constants we determined, and the levels of fluorescence recovery, for the various strains are summarized in **Table 2**.

**Table 2:** Summary of FRAP experiment analysis.

Strain	Activity^a^	D^b^ (µm^2^ s^−1^)	SD^c^	Tukey's test^d^	n^e^	Recovery^f^ (%)	n^g^
PAO1(pMMB*-mcherry*)	NA	3.71	0.53	a	4	100	
*pvdS-yfp*	**	1.08	0.47	b	55	100	
*pvdA-yfp* in spot	**	0.58	0.12	c	24	100	
*pvdA-yfp* no spot	**	0.49	0.11	cf	13	100	
*pvdA-yfp* out spot	**	0.16	0.06	eg	25	100	
*fpvF-mcherry*	**	0.15	0.06	gh	71	100	
*mcherry-pvdQ*	**	0.38	0.18	df	79	100	
*pvdQ-mcherry*	**	0.23	0.11	eh	74	100	
*tonB-mcherry*	**	0.06	0.03	g	28	10 +/– 5	87
*tonB-mcherry* + PVD-Fe	**	0.05	0.02	dgh	3	35 +/– 9	4
*mcherry-pvdT*	**	0.003	0.001	gh	3	33 +/– 8	11
*mcherry-opmQ*	-	0.11	0.06	gh	10	32 +/– 1	13

a Fusion protein activity relative to the wild-type activity (** : full activity ; * : partial activity ; – : corresponding to the activity in the deleted strain).

b Median diffusion coefficient determined as described in the “Materials and Methods” section.

c Standard deviation of the diffusion coefficient.

d Results of analysis of variance (ANOVA) for the diffusion coefficients and where significant (p<0.001), conditions were separated using Tukey’s test (P<0.05) [65].

e Sample size for D evaluation.

f Mean fluorescence recovery (in %) evaluated from recovery profiles with the associated standard deviation, as described in the “Materials and Methods” section.

g Sample size for standard deviation of mean fluorescence recovery.

NA Not Applicable.

We first focused on protein diffusion in the cytoplasm using eYFP-labeled PvdA (PVD biosynthesis enzyme [6]) and PvdS (sigma factor). Starting from uniformly distributed PvdA-eYFP at the beginning of growth, patches of fluorescence appeared from the mid-exponential phase and developed such that they made up the majority of the population during the stationary phase [6]. First, only cells with a uniform fluorescence distribution were photobleached (**Figure 1**). A full fluorescence recovery after photobleaching was observed for both *pvdA-yfp* and *pvdS-yfp*, indicating that the proteins are completely mobile (**Table 2**). The fusion protein with the highest diffusion coefficient was PvdS-eYFP (1.08±0.53 µm^2^ s^−1^; Figure 2C). Homogeneously distributed PvdA-eYFP had a lower diffusion coefficient (0.49±0.11 µm^2^ s^−1^) (**Table 2**, *pvdA-yfp* no spot row). Kumar et al. used various cytoplasmic fluorescent fusion proteins, and reported that diffusion in *E. coli* was primarily governed by the molecular mass (MM) of the fusion protein, according to the relation D ∼ (MM) ^−2^ [47]. Nenninger et al. observed the same size dependence of diffusion using multimers of GFP in *E. coli* [48]; however, they reported that free cytoplasmic diffusion followed the Einstein-Stokes equation for proteins below 110 kDa [48]. Approximation of a protein radius as being proportional to the volume and thus its molecular weight (MM) leads in this case to the relation D ∼ (MM) ^−1/3^. Nenninger *et al*. argued that this discrepancy, arises from slowing due to specific interactions of the fused proteins with cytoplasmic partners or the inner membrane [48]. To assess the size dependence of the diffusion coefficients, we used as the reference for free cytoplasmic diffusion a PAO1 strain harboring a plasmid encoding a cytoplasmic mCHERRY fluorescent protein expressed under the control of the *pvdA* promoter (PAO1(pMMB-*mcherry*)). In this case, recovery after photo-bleaching was very rapid and the diffusion coefficient we calculated for cytoplasmic mCHERRY was 3.71±0.53 µm^2^ s^−1^ (**Table 2**). Based on this free diffusion value, the Einstein-Stokes equation predicts diffusion coefficients of 2.58 for PvdA and 3.08 µm^2^ s^−1^ for PvdS which is faster that the measured values 0.49±0.53 µm^2^ s^−1^ and 1.08±0.53 µm^2^ s^−1^, respectively. Our values fit well with the relationship between D and the MM described by Kumar et al. [47]: this model leads to diffusion coefficients of 0.47 for PvdA and 1.25 µm^2^ s^−1^ for PvdS, which are very close to our experimental data, respectively. Our experiments thus indicate that both PvdS and unpatched PvdA diffuse freely in the cytoplasm, but that their diffusion might be slowed down by specific interactions.

### Association of PvdA at the old cell pole affects its diffusion but not its mobility

FRAP experiments were used to evaluate the stability of the fluorescent patches of PvdA at the old cell pole of the bacteria, and the diffusion of PvdA. Two approaches can be used for photo-bleaching a cell that presents a spot at one pole – extinction of the spotted pole or of the unspotted pole. The first option results in a bacterium with half the homogeneously distributed fluorescence, which is the same as photo-bleaching a cell with no fluorescent patch. In this case, the diffusion coefficient was found to be 0.58±0.12 µm^2^ s^−1^ (**Table 2**, *pvdA-yfp* in spot row). As previously, there was full recovery, yielding bacteria with homogeneously distributed fluorescence (**Table 2**). Thus, the former presence of a spot did not affect the diffusion of the pool of free cytoplasmic PvdA, which was the same as that measured for cells with no fluorescence patches (0.49±0.11 µm^2^ s^−1^, **Table 2**, *pvdA-yfp* no spot row). Bleaching the unspotted pole allows analysis of the characteristics of the patch of protein. In this case, we observed dissolution of the fluorescent patch, leading to full recovery and a uniform distribution (**Table 2**). This indicated that inclusion of PvdA protein in this structure was not irreversible, and that the population of the patch was dynamic. The diffusion coefficient we obtained for PvdA in patches, 0.16±0.06 µm^2^ s^−1^ (**Table 2**, *pvdA-yfp* out spot row), one third of that for the uniformly distributed population of protein molecules. Thus, the structure appearing as fluorescence patches involves interactions not affecting the pool of free cytoplasmic PvdA, although the mobility of the protein is maintained irrespective of its localization in the cell.

### The periplasmic binding protein FpvF is less dynamic than the periplasmic enzyme PvdQ involved in PVD biosynthesis

PvdQ is an N-terminal nucleophile hydrolase (Ntn-hodrolase) that is produced as a precursor protein, and undergoes autoproteolytic cleavage once in the periplasm [11]. This event leads to the formation of two subunits, a small α- and a heavier β-chain, coming from the N-terminal and C-terminal parts of the precursor, respectively. The two chains interact to form the active site of the protein. Strains *mcherry-pvdQ* and *pvdQ-mcherry* thus produced fully active and stable fusions with the α- and the β-chain, respectively [6]. In FRAP experiments, both strains showed full recovery of the fluorescence, indicating that PvdQ is fully mobile (**Table 2**). The diffusion coefficients were 0.38±0.18 for mCHERRY-PvdQ and 0.23±0.11 µm^2^ s^−1^ for PvdQ-mCHERRY (**Table 2**). Tukey’s test indicated a significant difference between these diffusion coefficients, but they were nevertheless sufficiently close to be consistent with the diffusion of the same protein.

Strain *fpvF-mcherry* (FpvF being one of the two periplasmic binding proteins of the FpvCDEF ABC transporter involved in PVD-Fe uptake) displayed full fluorescence recovery indicating the FpvF-mCHERRY proteins were fully mobile (**Table 2**); however, the diffusion coefficient was 0.15±0.06 µm^2^ s^−1^ which is lower than that for PvdQ.

### TonB inner membrane protein is less mobile than the PvdRT-OpmQ efflux pump but mobility of TonB is increased in the presence of PVD-Fe

The mCHERRY-PvdT fusion, involving the inner membrane anchor of the efflux pump PvdRT-OpmQ, yielded satisfactory fluorescence signal for FRAP whereas the fusion with the periplasmic adaptor protein, mCHERRY-PvdR, did not (**Figure 1** **)**. After photobleaching *mcherry-pvdT*, only 33±8% of the fluorescence was recovered over the 20 s of observation, indicating that 67% of the proteins are immobile (**Table 2**). Because of the small mobile fraction of mCHERRY-PvdT and the weak initial level of fluorescence, determination of the diffusion coefficient was limited by the small variation of the Fourier amplitudes; nevertheless, a diffusion coefficient of 0.003±0.001 µm^2^ s^−1^ was obtained, revealing that the mobile fraction was not dynamic (**Table 2**). The N-terminal fusion of mCHERRY to OpmQ resulted in a decrease of PVD secretion (**Figure S4D in File S1**) and a higher diffusion coefficient (0.11±0.06 µm^2^ s^−1^) than for mCHERRY-PvdT; the mobile fraction, however, was the same as in *mcherry-pvdT* (**Table 2**).

**Figure 3:**
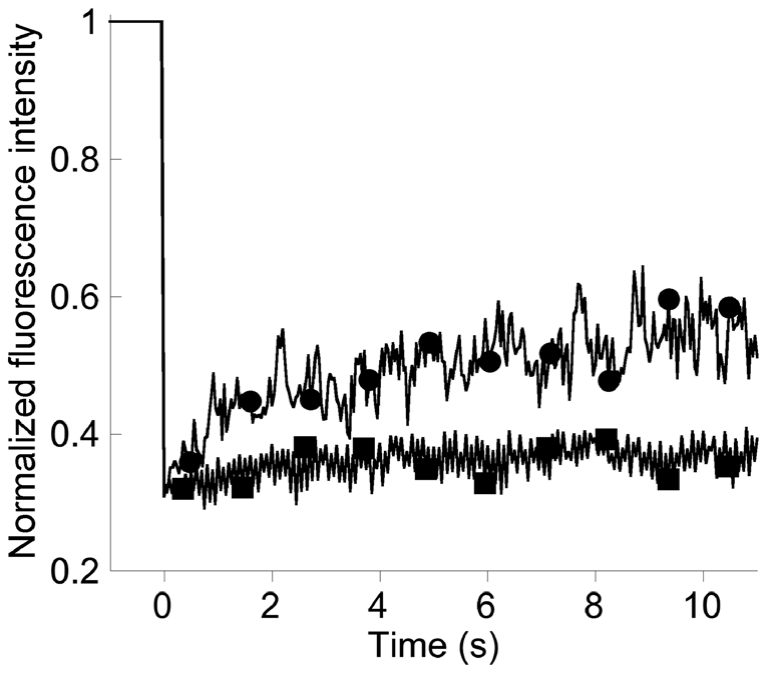
Recovery curves for TonB-mCHERRY in the absence (•) or presence (▪) of PVD-Fe. The fluorescence intensity from FRAP stream experiments were normalized using the FRAP Analysis plugin in Image-J_NIH_ software [66]. The averaged recovery profiles for TonB-mCHERRY (▪, n = 87) and TonB-mCHERRY after addition of 10 µM PVD-Fe (•, n = 4) are presented.

The inner membrane protein TonB, part of the TonB-ExbB-ExbD complex, plays a crucial role in ferri-siderophore uptake by energizing the outer membrane transporters [22], [49] (for a review, see [50]). In TonB-mCHERRY photobleaching experiments, only 10±5% of the fluorescence was recovered within 20 s, evidence that a large proportion of TonB proteins were immobile; the diffusion coefficient of the mobile fraction of TonB-mCHERRY was 0.06±0.03 µm^2^ s^−1^ (**Figure 2C**). We tested whether activation of the TonB machinery affected the dynamics of the protein. Just prior to FRAP data acquisition, we added PVD-Fe complex to a final concentration of 10 µM, to the strain *tonB-mcherry* samples. In these conditions, there was a significant increase in the mobile fraction of TonB-mCHERRY, with the fluorescence recovery rising to 35±9% (**Table 2** **,** and see averaged recovery curves in **Figure 3** **)**; the diffusion coefficient of the mobile fraction was 0.05±0.02 µm^2^ s^−1^, similar to the value in the absence of PVD-Fe (**Table 2**). Thus, activation of the TonB-ExbB-ExbD complex leads to an increase in the mobile fraction of the proteins but no increased diffusion.

## Discussion

We report the use of fluorescent labeling of proteins involved in the PVD pathway in *P. aeruginosa*. This is the first such analysis and it revealed that, although the cytoplasmic PVD biosynthesis machinery exhibited a high degree of organization [6], all the other proteins studied (those involved in periplasmic maturation of PVD, PVD secretion, iron import by PVD and the regulation of gene expression, **Scheme 1**) were uniformly distributed throughout the cell compartment in which they are found (**Figure 1**). Thus, the distributions of TonB and FpvF indicate that iron can be taken up by PVD at any position on the bacterial surface and periplasm. PVD assembly in the cytoplasm starts at the old pole of the bacterial cell [6], it ends in the periplasm with the PVD [7] and the enzymes involved in the last step of PVD maturation [6] diffusing throughout this cell compartment. The distribution of the efflux pump is consistent with the uniform distribution of newly synthesized PVD in the periplasm [7]; diffusing siderophores all around the bacteria presumably maximizes the efficiency of iron acquisition.

We also, for the first time, determined the diffusion characteristics of *P. aeruginosa* proteins and could compare them to the better studied model organism *E. coli*. We found that diffusion in the cytoplasm or periplasm is slower in *P. aeruginosa*, probably due to *P. aeruginosa* being smaller (around 1.5 µm) than *E. coli* (2 µm) but having a larger genome (6.3×10^6^ bp and 4.6×10^6^ bp, respectively). As in *E. coli*, the cytoplasm and the periplasm are fluid environments, with diffusion in cytoplasm being twice as fast as that in the periplasm. The PvdQ protein is not known to have any binding partners or to associate with the membrane; by adding a fluorescent tag, we constructed a 90 kDa fusion protein similar in size to the cytoplasmic PvdA-eYFP. The diffusion of this PvdQ fusion in the periplasm was twice as slow as that of PvdA-eYFP in the cytoplasm. In *E. coli*, the free diffusion of periplasmic GFP is 3-fold slower than that in the cytoplasm for cells grown in LB media [51] but the difference between the compartments varies depending on the growth medium and the osmolality [52]. Proteins anchored in inner or outer membranes in the two species seem to share similar diffusion characteristics. Although these findings require confirmation by more informative approaches, they provide a basis for the comparison of protein dynamics between these two species.

PvdS is a cytoplasmic protein, and was uniformly distributed suggesting that it most probably diffuses freely in this cell compartment. Tiburzi *et al*. reported that the alternative sigma factor PvdS is mostly present in the cytoplasm free from any RNA polymerase complex [53]. Consistent with this, the pattern of diffusion of RNA polymerase complexes in *E. coli* are dissimilar from that of PvdS (Table 2) with an immobile fraction of 47% and slow diffusion (0.22±0.16 µm^2^ s^−1^) for the mobile fraction [54]. In addition to being involved in transcription, PvdS can also bind to FpvR, its cognate inner membrane anti-sigma, to regulate its activity [31]. We did not detect PvdS-eYFP bound to the membrane (**Figure S1C in File S1**) in our iron-limited growth conditions, so the diffusion we observed can be attributed mostly to PvdS free from FpvR complexes. No other cytoplasmic protein has been reported to bind to PvdS. Therefore, most PvdS in *P. aeruginosa* cells grown in iron-limited media presumably diffuses freely in the cytoplasm. This high abundance of PvdS in the cytoplasm probably allows the alternative sigma factor to compete with RpoD and promote the transcription of the genes it regulates [53]. 

Similarly, the diffusion of the cytoplasmic PvdA protein that is not incorporated into patches at the old cell pole shows the same size dependency as PvdS. This suggests that, when present in the cytoplasm, PvdA is not involved in protein complexes. However, the diffusion of PvdA in patches at the old cell pole was consistently three fold slower presumably reflecting interactions with the cytoplasmic membrane and partners in the cytoplasmic biosynthesis of PVD. The association of PvdA at the old cell pole is however completely reversible: full recovery was observed in FRAP experiments. PVD biosynthesis thus appears to be a highly dynamic process with PvdA and probably other partners switching between patches at the old cell pole, which may correspond to the siderophore assembly chain [6], and the cytoplasmic compartment.

Diffusion of the periplasmic enzymes PvdQ and FpvF also illustrated slow-downs due to complex formation with substantially slower diffusion of the periplasmic binding protein FpvF than PvdQ. No protein partner is known for PvdQ. By contrast, the slower diffusion of FpvF probably reflects its interactions with its cognate inner membrane partners FpvD and FpvE, and the second periplasmic binding protein FpvC of this FpvCDEF ABC transporter [24]. Additional interactions that remain to be identified for FpvF may also contribute to its slow diffusion. Interactions between TonB and the periplasmic binding proteins FhuD of the ferrichrome iron uptake pathway and BtuF of the vitamin B_12_ import system in *E. coli* have been reported [55], [56].

We also report the first description of diffusion of a tripartite efflux pump, PvdRT-OpmQ. The diffusion pattern of mCHERRY-PvdT (33% of the protein being mobile with a diffusion constant of 0.003±0.001 µm^2^ s^−1^) is rather unusual among inner membrane proteins: most such proteins are fully mobile as reported for various fluorescent inner membrane fusion proteins in *E. coli* [36], [41], [47], [51]. As mCHERRY-PvdT is associated with the membrane fraction (**Figure S4B in File S1**) and thus probably interacts strongly with PvdR and OpmQ, this diffusion data may represent the entire PvdRT-OpmQ efflux system. Indeed, both mCHERRY-PvdT and mCHERRY-OpmQ have equivalent mobile fractions and the diffusion coefficient of mCHERRY-PvdT is very close to the 0.006±0.002 µm^2^ s^−1^ determined by single molecule approach for the outer membrane protein OmpF in *E. coli* [39]. This value is however one order of magnitude below those of the *E. coli* maltose outer membrane transporter LamB [38] or the vitamin B12 outer membrane transporter BtuB [39]. Two studies on LamB in *E. coli* report that 40% of the population is immobile [37], [38]. To summarize, all components of PvdRT-OpmQ seem to be tightly associated and only 30% of the population is mobile whereas the diffusion coefficient we determined is consistent with an outer membrane component limiting the mobility of the efflux pump as a whole.

One of the major findings of this work concerns TonB, the central player in ferri-siderophore uptake by the outer membrane transporter FpvA. The way TonB activates ferri-siderophore outer membrane transporters has been the subject of many investigation the last years but remains unresolved [57], [58]. We found that most TonB protein molecules (90%) are immobile, which is unusual for an inner membrane protein. TonB is embedded in the inner membrane, and the TonB machinery is composed of TonB, ExbB and ExbD probably with the following stoichiometry: 1:7:2, respectively [59]. The N-terminus of TonB is inserted into the cytoplasmic membrane and its C-proximal region interacts, in the periplasm, with the region called the TonB box of the outer membrane transporters of siderophores [18], [20]. ExbB spans the inner membrane three times and most of the protein is in the cytoplasm, whereas the organization of ExbD is similar to that of TonB. When TonB interacts with an outer membrane transporter, these bridged complexes between the outer membrane and the inner membrane proteins may become immobile, preventing diffusion in either membrane. Consistent with this hypothesis, the diffusion of TonB is similar to that of BtuB transporter in *E. coli* (a TonB-dependent transporter involved in vitamin B12 uptake) comforting the view that TonB restricts the diffusion of its outer membrane transporters [39]. Another possible explanation of the immobility of TonB, is that the TonB machinery is anchored to a static scaffold within or underneath the cell membrane. Both ExbD and ExbB are cross-linked by formaldehyde *in vivo* to unidentified proteins [60]–[62], which may be partners of a protein organization of this type. In the presence of PVD-Fe, TonB mobility was substantially higher whereas the diffusion coefficient for the mobile fraction remained unchanged. This increased TonB mobility in the presence of PVD-Fe may be a consequence of the loss of the interaction with its outer membrane partner, FpvA, or of TonB transition to another activated state, assisted by ExbB and/or ExbD. Our analyses provide novel important data and insight, but further investigations focused on the TonB machinery using the same approach would help decipher the apparently complex mechanism of energy transfer. It is also interesting to observe that, upon activation, the mobile fraction of TonB-mCHERRY is comparable to that of mCHERRY-PvdT, a component of the efflux pump that is believed to be activated at any time and which is also in complex with an outer membrane protein (OpmQ).

In conclusion, we report the first visualization of an entire siderophore pathway by fluorescent microscopy in living cells, and the dynamics of the proteins involved. We describe various different mobilities, probably depending on the protein interaction networks involving the proteins studied. The data clearly show that TonB is immobile in the basal state, and its mobility is increased in the presence of PVD-Fe; these are some of the first insights into the dynamics of the complex energy transfer mechanisms between TonB in the inner membrane and the outer membrane transporters of siderophores. Of more general interest, this work has also yielded the first data about protein diffusion in the various cell compartments of *P. aeruginosa*: cytoplasmic and periplamic diffusion appeared slower than in *Escherichia coli* but membrane proteins seemed to behave similarly in the two species.

## Supporting Information

File S1 Contains the following: Table S1: Oligonucleotides used in this study. Table S2: Plasmids used in this study. Figure S1: Phenotypic characterization of *pvdS-yfp*. Figure S2: Phenotypic characterization of *fpvF-mcherry*. Figure S3: Phenotypic characterization of *tonB-mcherry*. Figure S4: Phenotypic characterization of *mcherry-pvdR, mcherry-pvdT*, *mcherry-opmQ* and *opmQ-mcherry*. Figure S5: Fluorescence microscopy analysis of fluorescent PAO1 expressing mCHERRY (strain PAO1(pMMB-*mcherry*)).
(DOC)
