# Supplementary material for: Deciphering Protein Dynamics of the Siderophore Pyoverdine Pathway in Pseudomonas aeruginosa
Source: PLoS One. 2013 Oct 30;8(10):e79111. doi: 10.1371/journal.pone.0079111 (PMC3813593; doi:10.1371/journal.pone.0079111)
Supplement: File S1 — Contains the following: Table S1: Oligonucleotides used in this study. Table S2: Plasmids used in this study. Figure S1: Phenotypic characterization of pvdS-yfp. Figure S2: Phenotypic characterization of fpvF-mcherry. Figure S3: Phenotypic characterization of tonB-mcherry. Figure S4: Phenotypic characterization of mcherry-pvdR, mcherry-pvdT, mcherry-opmQ and opmQ-mcherry. Figure S5: Fluorescence microscopy analysis of fluorescent PAO1 expressing mCHERRY (strain PAO1(pMMB-mcherry)). (DOC) [file pone.0079111.s001.doc]

**SUPPLEMENTAL MATERIALS**

**Deciphering protein dynamics of the siderophore pyoverdine pathway in *Pseudomonas aeruginosa***

Laurent Guillon^1^, Stephan Altenburger^2^, Peter L. Graumann^2^, Isabelle J. Schalk^1^

^1^UMR 7242, Université de Strasbourg-CNRS, ESBS, Blvd Sébastien Brant,

F-67413 Illkirch, Strasbourg, France

^2^SYMMIKRO, LOEWE Center for Synthetic Microbiology, and Department of Chemistry, University of Marburg, Germany.

**SUPPLEMENTAL Materials and Methods**

***Mutant construction*.** All enzymes for DNA manipulation were purchased from Fermentas and used according to the manufacturer’s instructions. *Escherichia coli* strain TOP10 (Invitrogen) was used as a host strain for all plasmids. PCR, using *iProof* polymerase (Bio-Rad), was used to amplify the genes encoding the fluorescent proteins from the following cDNAs: pCDNA3.1/mCHERRY for mCHERRY, pCDNA3.1/eYFP for eYFP and pCDNA3.1/eGFP for eGFP. The DNA fragments from *Pseudomonas* used for cloning were amplified from the PAO1 genomic DNA. The general procedure consists in the cloning in pME3088 suicide vector of a construct composed of the fluorescent protein encoding DNA flanked by the 700bp upstream and downstream regions relative to the insertion site. Mutations in the chromosomal genome of *P*. *aeruginosa* were generated by transferring suicide vectors from *E*. *coli* S17-1 to the PAO1 strains and integration of the plasmids into the chromosome, with selection for tetracycline resistance. A second crossing-over event excising the vector was achieved by enrichment for tetracycline-sensitive cells to generate the corresponding mutants. All gene replacement mutants were checked by PCR.

The primers used are listed in Table S1. The names of the primers start with the name of the corresponding strain. For amplification of the 700 bp upstream region relative to the insertion site, primers with the UF and UR extensions were used as forward and reverse primer, inserting restriction sites for ligation into pME3088 and to the 3’ end of the fluorescent protein encoding DNA, respectively. The 700 bp downstream region was amplified with forward and reverse oligonucleotides with the DF and DR extensions, inserting restriction sites for ligation to the 5’ end of the fluorescent protein encoding DNA and into pME3088, respectively. The fluorescent protein encoding DNA was amplified with oligonucleotides named Fluo (3’ and 5’ ends of all fluorescent protein encoding DNA are identical), followed by the name of the restriction enzyme and F and R extensions, for forward and reverse. These primers insert restriction sites compatible with those of UR and DF primers previously used, respectively. The two first PCR products from PAO1 genomic DNA were single digested by the restriction enzymes required for ligation with the fluorescent protein encoding DNA. The PCR product coding for the fluorescent protein was double digested by the compatible restriction enzymes. Following mixing in equimolar amounts and ligation, the mixture was used as a template for further amplification with the external oligonucleotides, with UF and DR extensions. The PCR product was double digested with the restriction enzymes allowing cloning into the pME3088 vector, linearized by the same enzymes.

For N-terminal insertion of proteins, the fluorescent protein encoding DNA was inserted right after the start codon except when a signal sequences is present. In such case, the insertion site was placed after the predicted protease cleavage site, e.g. after residues 25 and 19 for PvdR and OpmQ, respectively. In C-terminal fusion, the insertion site was placed before the stop codon.

All the plasmids used in this study are listed in Table S2.

*pMMB190-PpvdA-mcherry (pLG3) construction*. The promoter region of *pvdA* was amplified by PCR from PAO1 genomic DNA using oligonucleotides PPvdA-BamHIF and PPvdA-XhoIR, inserting restriction sites BamHI and XhoI restriction sites, respectively. The DNA encoding sequence of mCHERRY was pCDNA3.1/mCHERRY using oligonucleotides mCHERRY-XhoIF and mCHERRY-HindIIIR, inserting XhoI and BamHI restriction sites, respectively. The two PCR products were digested by XhoI and ligated. The ligation mixture served as a template for further amplification by PvdA-BamHIF and mCHERRY-HindIIIR. Following digestion by BamHI and HindIII, the PCR product was cloned into pMMB190, linearized by the same enzymes.

*Growth and PVD production.* Growth and PVD production were monitored in a Tecan microplate reader (Infinite M200, Tecan) using the growth conditions described previously . Each measurement is a mean over 6 replicates. A blank made of minimal media was subtracted to raw data and OD_400nm_ values (corresponding to PVD production) at a given time were divided by the corresponding OD_600nm_ values for normalization.

*Immunoblot analysis.* Immunoblot analysis were carried out as described previously with the following antibodies dilutions: anti-FpvC antibodies (3:2000 dilution), anti-FpvF antibodies (3:2000 dilution), anti-TonB antibodies (1:1000 dilution), anti-OpmQ antibodies (1:1000 dilution), anti-GFP antibodies (1:10000 dilution, Roche), anti-DsRed antibodies (1:1000 dilution, Ozyme), IRDye 800CW donkey anti-rabbit IgG (1:10000 dilution, Clinisciences) and 700 donkey anti-mouse IgG secondary antibody (1:10000 dilution, Clinisciences). Antibody binding was detected with an Odyssey Imager (LI-COR Biosciences).

*Cell fractionation*. Cell fractionation was carried out as described previously . The fluorescence in each fractions was measured in a Tecan microplate reader (Infinite M200, Tecan) using the following excitation/emission wavelengths: 400 nm/450 nm, 500 nm/540 nm and 570 nm/610 nm for PVD, eYFP and mCHERRY, respectively.

*Iron uptake assays* PVD-^55^Fe (0.25 Ci mmol^–1^) was prepared as previously described , with a 4-fold excess of PVD over iron. The uptake assays were carried out as previously described in the presence of 100 nM or 500 nM of PVD-^55^Fe for TonB or FpvF mutant strains characterization, respectively. As a negative control, the experiment was repeated with cells that had been previously treated with 200 μM CCCP, which is a protonophore that inhibits iron uptake .

*Periplasmic PVD accumulation analysis.* Samples were prepared as described in Material and Method section of the main text. Epifluorescence images of the efflux pump mutant strains were acquired on a Nikon 50i (objective: CFI Achroplan 100× A ON 1.25 DT 0.18) microscope equipped with a numeric 12 bits DS-Fi1 camera. For PVD imaging, a BV-2A filter (excitation 420 ± 20 nm, emission 470 nm longpass, dichroic filter 455 longpass) was used keeping the same illumination for all compared strains. Images were captured using imaging software NIS elements.

| **Oligonucleotides** | **Sequence (5’ to 3’)** |
| --- | --- |
| PPvdA-BamHIF | AATAGGATCCGCATGCGCGAATGGG |
| PPvdA-XhoIR | GTTGCTCGAGTCATTTCCAGTTCCTC |
| mCHERRY-XhoIF | AAATCTCGAGTGAGCAAGGGCGAG |
| mCHERRY-HindIIIR | CTGCAAGCTTACTTGTACAGCTTCGTC |
| PvdS-YFP-UF | AAAGAATTCTGCGGACCATTCACGAATAAAG |
| PvdS-YFP-UR | TTTTCTAGATCTGCGGGCGCTGAGATGG |
| PvdS-YFP-DF | AAAGTCGACTGACGGCGGCGAGCATTC |
| PvdS-YFP-DR | TTTAAGCTTCACCACCTGTTGCAGCAG |
| FpvF-mCHERRY-UF | AAAGAATTCCAGGCCTCGTTCTTTGCTG |
| FpvF-mCHERRY-UR | TTTTCTAGAGCCCTTACCGGCCAGCAGC |
| FpvF-mCHERRY-DF | AAACTCGAGTGAGCCGCGCCTCGTAAC |
| FpvF-mCHERRY-DR | TTTAAGCTTGCGAGCCTGGCGCAGCAAC |
| TonB-mCHERRY-UF | AAGAATTCATCATCGGTGCGCTGGTGTGG |
| TonB-mCHERRY-UR | AAATCTAGAGCGGCGCTTCTCGATCTTG |
| TonB-mCHERRY-DF | AAAATCGATTGAGGTTCGCCGACAACG |
| TonB-mCHERRY-DR | CTGCAAGCTTTCGACAGCTGGTGGG |
| mCHERRY-PvdR-UF | AAAGAATTCACATCCGCCGGGTTTCCC |
| mCHERRY-PvdR-UR | TTTTCTAGAGGCGGCCAGGGCGATCAG |
| mCHERRY-PvdR-DF | AAAATCGATTGGCAGGCCTATCCGTTCC |
| mCHERRY-PvdR-DR | TTTAAGCTTCTTGCCGGTCCAGCGCC |
| mCHERRY-PvdT-UF | AAAGAATTCGATCGAGATGTACCAGGC |
| mCHERRY-PvdT-UR | TTTTCTAGACATCAGCTGTCGCTGCCG |
| mCHERRY-PvdT-DF | AAAATCGATGAAAACGCCACGCAACCC |
| mCHERRY-PvdT-DR | TTTAAGCTTGTTGGCGCTGTCGCTGAC |
| mCHERRY-OpmQ-UF | AAAGAATTCGAGAAAGGCTCCAGCTCC |
| mCHERRY- OpmQ-UR | TTTTCTAGAGCCGCAGGCGCCGAGCAG |
| mCHERRY- OpmQ-DF | AAAATCGATAGCACGCCGGCGCCCC |
| mCHERRY- OpmQ-DR | TTTAAGCTTGCCGATCAGGGTGGCCAG |
| Fluo-XbaIF | AAATCTAGAGTGAGCAAGGGCGAGGAG |
| Fluo-SalIR | AAAGTCGACCTTGTACAGCTCGTCCAT |
| Fluo-XhoIR | TTTCTCGAGCTTGTACAGCTCGTCC |
| Fluo-ClaIR | AAAATCGATCTTGTACAGCTCGTCCAT |

**Table S1: Oligonucleotides used in this study.** Restriction sites are underlined.

| **suicide vector and mutator** | **Relevant characteristics** | **Source or reference** |
| --- | --- | --- |
| pME3088 | suicide vector; Tc^R^ ; ColE1 replicon ; *Eco*RI *Kpn*I *Dra*II *Xho*I *Hin*dIII polylinker |  |
| pMMB190 | Ap^R^, pMMB66EH, tac promoter, *LacZα* |  |
| pLG3 | pMMB190-*PpvdA-mcherry* | this study |
| pLG4 | pME3088 **Δ***pvdS* | this study |
| pLG6 | pME3088 *pvdS-yfp* with 700 bp flanking region relative to *yfp* | this study |
| pLG8 | pME3088 *fpvF-mcherry* with 700 bp flanking region relative to *mcherry* | this study |
| pLG10 | pME3088 *tonB-mcherry* with 700 bp flanking region relative to *mcherry* | this study |
| pLG11 | pME3088 *mcherry-pvdR* with 700 bp flanking region relative to *mcherry* | this study |
| pLG13 | pME3088 mcherry-*pvdT* with 700 bp flanking region relative to *mcherry* | this study |
| pLG15 | pME3088 *mcherry-opmQ* with 700 bp flanking region relative to *mcherry* | this study |

**Table S2: Plasmids used in this study.**

**Phenotype characterization of the fusion proteins**

***Phenotype characterization of pvdS-yfp*.** The sigma factor PvdS activates the transcription of all PVD genes except *fpvA*. We generated strains expressing C-terminal fusion of PvdS with eYFP fluorescent protein, *pvdS-yfp* strains. Expression of the fusion protein was checked by immunoblot analysis on whole cells from wild type PAO1, PAO1*pvdS* (*pvdS* deletion mutant) and *pvdS-yfp*. Anti-GFP antibodies that detect all variants of *Aequorea victoria* GFP were used for detection (**Figure S1A**). As expected, no signal was detected in PAO1 or PAO1*pvdS*. Insertions yielded a single band above the 35 kDa marker band whereas fusion of PvdS with eYFP would generate 47 kDa proteins. However, purified PvdS was previously shown to migrate at an apparent mass of 28 kDa whatever under its native or FLAG-tagged form due to anomalous migration of the sigma factor . This may explain the low shift between eGFP and its fusions with PvdS (**Figure S1A**).

Bacterial growth as well as PVD production were monitored for PAO1, PAO1*pvdS* and *pvdS-yfp* (**Figure S1B**). The strain *pvdS-yfp* grew and produced PVD in the same extent as PAO1.

The cytoplasmic localization of the fusion protein was assessed by cellular fractionation on *pvdS-yfp* cells (**Figure S1C**). As previously, PVD was used as a control for cellular fractionation success and was predominantly found in the periplasmic fraction . About 65 % of the eYFP fluorescence was detected in the cytoplasmic fraction for *pvdS-yfp* and 35 % within the periplasm (**Figure S1C**). The high level of fluorescence of eYFP detected in the periplasm must arise from a contamination by PVD fluorescence since these two fluorophores have close spectral properties. Indeed, PvdS does not contain any signal sequence to allow its transport into the periplasm. The sigma factor could however be found associated with its anti-sigma factor FpvR that is inserted into the inner membrane. We only found 1 % of eYFP fluorescence in the membrane fraction suggesting that PvdS-eYFP is predominantly cytoplasmic (**Figure S1C**).


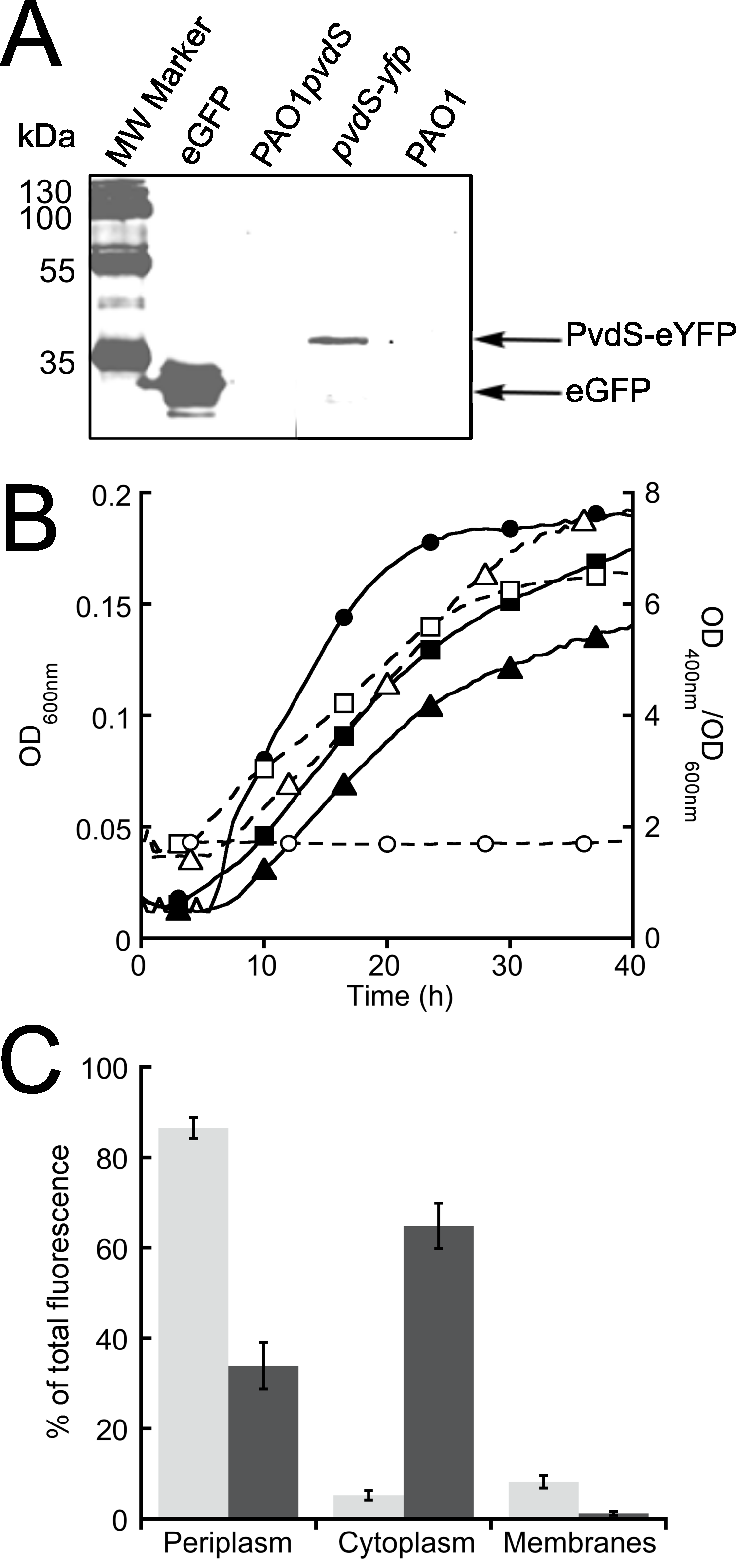


**Figure S1: Phenotypic characterization of *pvdS-yfp*.** (**A**) Immunoblot analysis of PAO1, PAO1*pvdS* and *pvdS-yfp* strains. The equivalent of 0.25 OD_600_ units of each strain were lysed in loading buffer, DNA digested by benzonase (1 U) and loaded onto SDS PAGE for protein separation. Proteins were blotted onto nitrocellulose membrane and eYFP protein was detected using anti-GFP antibodies. Molecular weight (MW) marker bands are indicated on the left. (**B**) The growth (solid lines, filled markers) and PVD production (dashed lines, empty markers) of PAO1 (◼), PAO1*pvdS* (⚫) and *pvdS-yfp* (▲) strains were monitored by OD_600_ and OD_400_ measurements over time. Iron-starved bacteria were inoculated in fresh minimal media and distributed in 96 wells plate. Measurements were carried out every 30 min in a Tecan microplate reader with shaking and incubation at 30°C. Every single curve is a mean over 6 replicates. PVD production (OD_400_) at a given time was normalized by corresponding OD_600_. Every single curve is a mean over 6 replicates. (**C**) Cellular fractionation of *yfp-pvdS* and *pvdS-yfp* strains**.** Fluorescence of PVD and eYFP were measured for equal volumes of the cytoplasmic, periplasmic and cell membranes fractions. The data are means of three independent experiments and normalized as percentages of total fluorophore fluorescence. White and dark gray columns represent PVD and mCHERRY, respectively.

***Phenotype characterization of FpvF-mcherry*.** FpvF is a periplasmic binding protein of the ABC transporter FpvCDEF involved in iron uptake by PVD . mCHERRY encoding DNA was inserted at the C-terminus of FpvF, generating *fpvF-mcherry*. mCHERRY protein folds efficiently in the periplasm and can therefore be used for the labeling of periplasmic proteins . The integrity of the fusion proteins was assessed by subjecting whole cells from wild type PAO1, PAO*fpvCDEF* (*fpvCDEF* deletion mutant) and *fpvF-mcherry* strains grown in iron minimal media to SDS-PAGE and western blot analysis using antibodies raised against FpvF or Ds-Red proteins (**Figure S2A**). In PAO1 strain, a single band above the 35 kDa marker band was detected with anti-FpvF (**Figure S2A**) antibodies, corresponding to FpvF proteins. Accordingly, this band was not present in the deletion strain. Consistent with stable fusion proteins expression of ~ 57 kDa in *fpvF-mcherry*, a unique band above the 55 kDa marker bands was detected with anti-FpvF antibodies (**Figure S2A**). The presence of fused mCHERRY was further confirmed by detection of the same bands in insertion strains using the anti-DsRed antibodies (**Figure S2A**).

The subcellular localization of the protein was further tested by subjecting the insertion strains to cellular fractionation, using PVD fluorescence as a control. Consistent with a periplasmic localization of the fusion proteins, we found 90 % of mCHERRY fluorescence in the periplasmic fraction in *fpvF-mcherry* strains (**Figure S2B**).

The growth and PVD production in iron-depleted media were not affected for the insertion mutants or the deletion strain PAO*fpvCDEF* compared to the PAO1 strain (**Figure S2C**). The deletion of the ABC transporter was shown to induce a 30 % iron uptake decrease when 500 nM of PVD-^55^Fe was added to cells grown in iron minimal media . In the same conditions, the strain *fpvF-mcherry* transported iron as efficiently as the wild-type PAO1 strain (**Figure S2D**).


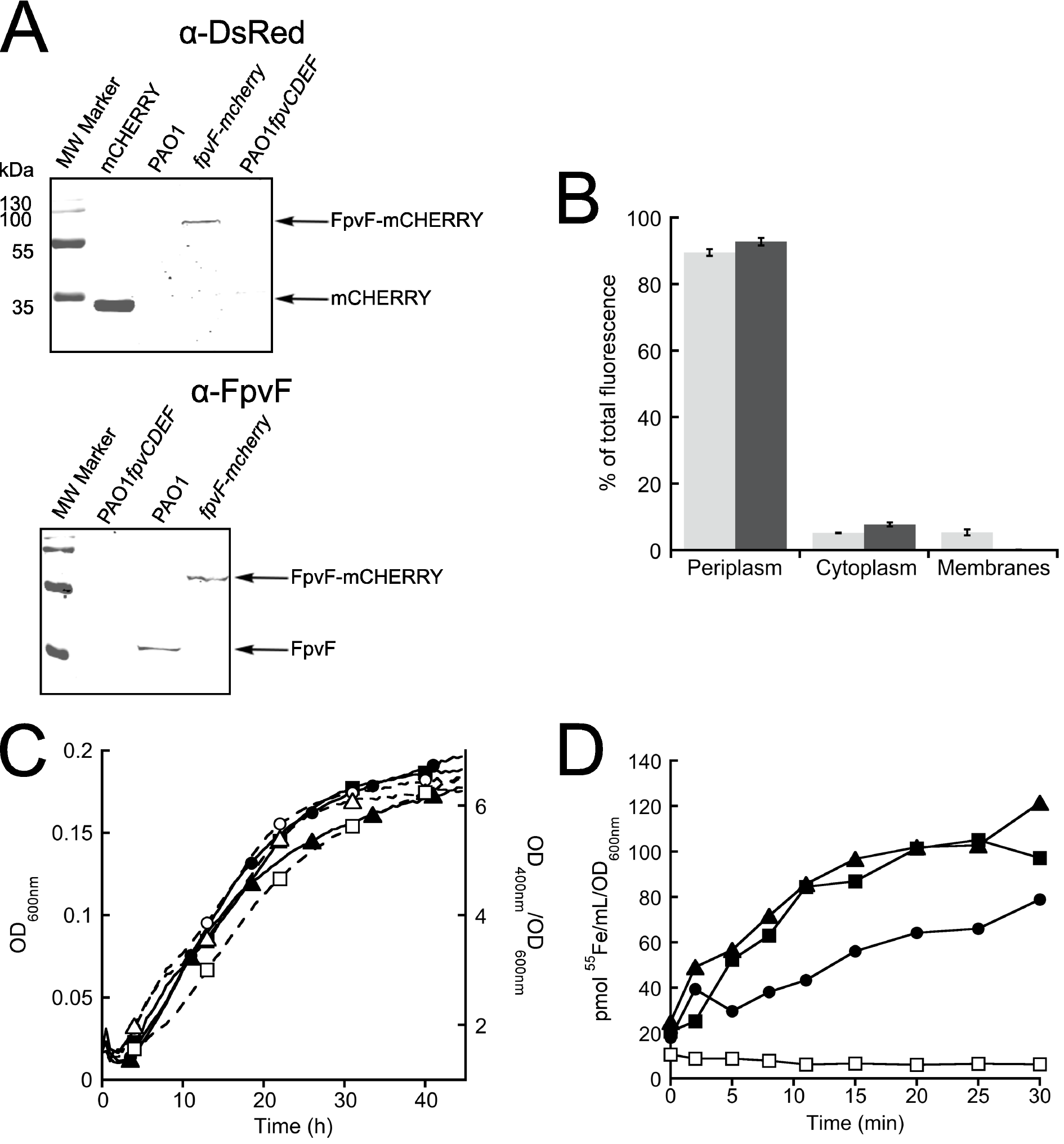


**Figure S2: Phenotypic characterization of *fpvF-mcherry*.** (**A**) Immunoblot analysis of PAO1, PAO1*fpvCDEF* and *fpvF-mcherry* strains. The equivalent of 0.25 OD_600_ units of each strain were lysed in loading buffer, DNA digested by benzonase (1 U) and loaded onto SDS PAGE for protein separation. Proteins were blotted onto nitrocellulose membrane before detection of mCHERRY by anti-DsRed (upper panel) or FpvF by anti-FpvF (lower panel) antibodies. Molecular weight (MW) marker bands are indicated on the left. (**B**) Cellular fractionation of *fpvF-mcherry* strain**.** Fluorescence of PVD and mCHERRY were measured for equal volumes of the cytoplasmic, periplasmic and cell membranes fractions. The data are means of three independent experiments and normalized as percentages of total fluorophore fluorescence. White and dark gray columns represent PVD and mCHERRY, respectively. (**C**) The growth (solid lines, filled markers) and PVD production (dashed lines, empty markers) of PAO1 (◼), PAO1*fpvCDEF* (⚫) and *fpvF-mcherry* (▲) strains were monitored by OD_600_ and OD_400_ measurements over time. Iron-starved bacteria were inoculated in fresh minimal media and distributed in 96 wells plate. Measurements were carried out every 30 min in a Tecan microplate reader with shaking and incubation at 30°C. Every single curve is a mean over 6 replicates. PVD production (OD_400_) at a given time was normalized by corresponding OD_600_. Every single curve is a mean over 6 replicates. (**D**) Time-dependent uptake of PVD-^55^Fe in *P. aeruginosa* PAO1 (◼), PAO1*fpvCDEF* (⚫) and *fpvF-mcherry* (▲) strains. Cells at an OD_600_ of 1 were incubated for 15 min at 37 °C in 50 mM Tris-HCl (pH 8.0) before the initiation of transport assays by the addition of 500 nM PVD-^55^Fe. Samples (100 μL) of the suspension were removed at various times and filtered, and the radioactivity retained was counted. The results are expressed as picomoles of PVD-^55^Fe transported per milliliter of cells at an OD_600_ of 1. The experiment was repeated with the protonophore CCCP at a concentration of 200 μM (PAO1 (🞎); data not shown for the other strains). CCCP inhibits all TonB-dependent transport in bacteria.

***Phenotype characterization of TonB-mcherry*.** The TonB-ExbB-ExbD complex transfers the proton-motive force of the inner membrane to the outer membrane transporter FpvA thanks to an interaction between the C-terminal of the TonB protein and a specific sequence in the N-terminal of the transporter called the TonB box (for a review, see ). The mCHERRY encoding DNA was inserted to the C-terminus of the TonB protein generating *tonB-mcherry* strains. The C-terminal end of TonB lays in the periplasm, therefore we chose the mCHERRY protein because it folds efficiently in the periplasm . The integrity of the TonB fusion proteins was checked by subjecting whole cells from PAO1, PAD08 (*tonB* deletion strain), *tonB-mcherry* strains grown in iron-depleted minimal media to SDS-PAGE electrophoresis followed by western blot analysis using anti-TonB antibodies. As expected, no TonB signal was detected in PAD08 deletion strain. In accordance with the predicted mass of TonB (36.8 kDa), a single band above 35 kDa is detected in PAO1 strain. The *tonB-mcherry* strain displayed a unique band between the 50 and 100 kDa marker bands (**Figure S3A**), which is in agreement with an insertion of mCHERRY protein in TonB that would generate a ~ 64 kDa fusion protein.

Unexpectedly, when trying to confirm the membrane bound state of the fusion protein, the *tonB-mcherry* strain exhibited lysis when cells where digested by lysozyme during the cellular fractionation. This may imply a possible membrane weakening upon fluorescent tag insertion in this strain.

No influence of fluorescent tag insertion on growth and PVD production in iron minimal media was observed (**Figure S3B**). We checked the ability of PAO1, the *tonB* deletion strain PAD08 and *tonB-mcherry* strains to transport ^55^Fe in the presence of PVD. As expected, deletion of *tonB* gene impeded the uptake of radiolabeled iron in complex with PVD (**Figure S3C**). The strain *tonB-mcherry* transported iron as efficiently as the PAO1 strain.


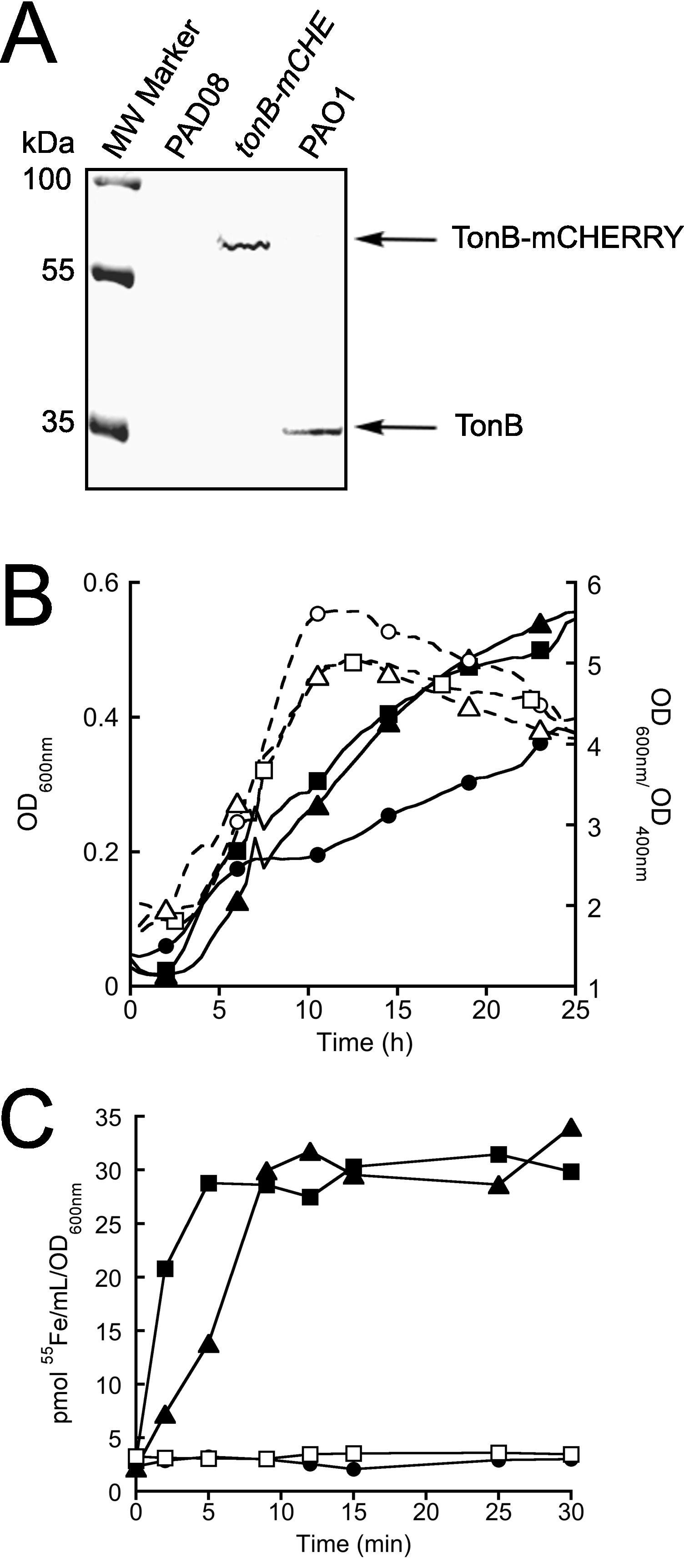


**Figure S3: Phenotypic characterization of *tonB-mcherry*.** (**A**) Immunoblot analysis of PAO1, PAD08 and *tonB-mcherry* strains. The equivalent of 0.25 OD_600_ units of each strain were lysed in loading buffer, DNA digested by benzonase (1 U) and loaded onto SDS PAGE for protein separation. Proteins were blotted onto nitrocellulose membrane and TonB was detected using anti-TonB antibodies. Molecular weight (MW) marker bands are indicated on the left. (**B**) The growth (solid lines, filled markers) and PVD production (dashed lines, empty markers) of PAO1 (◼), PAD08 (⚫) and *tonB-mcherry* (▲) strains were monitored by OD_600_ and OD_400_ measurements over time. Iron-starved bacteria were inoculated in fresh minimal media and distributed in 96 wells plate. Measurements were carried out every 30 min in a Tecan microplate reader with shaking and incubation at 30°C. Every single curve is a mean over 6 replicates. PVD production (OD_400_) at a given time was normalized by corresponding OD_600_. Every single curve is a mean over 6 replicates. (**C**) Time-dependent uptake of PVD-^55^Fe in *P. aeruginosa* PAO1 (◼), *tonB* deletion strain PAD08 (⚫), and *tonB-mcherry* (▲) strains. Cells at an OD_600_ of 1 were incubated for 15 min at 37 °C in 50 mM Tris-HCl (pH 8.0) before the initiation of transport assays by the addition of 100 nM PVD-^55^Fe. Samples (100 μL) of the suspension were removed at various times and filtered, and the radioactivity retained was counted. The results are expressed as picomoles of PVD-^55^Fe transported per milliliter of cells at an OD_600_ of 1. The experiment was repeated with the protonophore CCCP at a concentration of 200 μM (PAO1 (🞎); data not shown for the other strains).

***Phenotype characterization of mcherry-PvdT, mcherry-PvdR and mcherry-opmQ*.** PvdRTOpmQ is the efflux pump involved in siderophore secretion and recycling in the extracellular media . We produced *Pseudomonas aeruginosa* strains expressing the inner membrane anchor PvdT, the periplasmic adaptor protein PvdR or the outer membrane channel OpmQ fused with mCHERRY protein at the N-terminus. To assess the integrity of the fusion proteins, a western blot analysis was conducted on whole cells using anti-DsRed antibodies (**Figure S4A**), which can detect the mCHERRY protein, and anti-OpmQ antibodies (**Figure S4A**) for the strains expressing mutant variant of the OpmQ protein. As expected, no signal for mCHERRY protein was detected in PAO1 or PAO1*pvdRTopmQ* (*pvdRTopmQ* deletion) strains. No signal was detected for N-terminal fusions with either PvdR, PvdT or OpmQ, *mcherry-pvdR*, *mcherry-pvdT* or *mcherry-opmQ* strains, respectively (**Figure S4A**), which can be due to antibody detection problems linked to the generation of these fusion proteins. When OpmQ detection was performed, a single band at ~ 50 kDa corresponding to OpmQ was detected in the PAO1 strain but not in the PAO1*pvdRTopmQ* deletion strain and no bands for *mcherry-opmQ* (**Figure S4A**).

The fluorescent content of PVD and mCHERRY *in vivo* in the bacterial cell compartments was checked by cellular fractionation (**Figure S4B**). Again the success of the fractionation was assessed taking advantage of the previously reported periplasmic localization of the PVD siderophore . Surprisingly, mCHERRY could be detected in all insertion mutant strains indicating antibodies detection problems in the aforementioned western blot analysis. For all strains, around 30 % of the total mCHERRY fluorescence was found in the cytoplasmic fraction (**Figure S4B**), which is probably related to synthesized proteins prior to their export as what has been previously observed with *mcherry-pvdQ* and *pvdQ-mcherry* strains . Consistent with their predicted localization, the mCHERRY fusions with the inner membrane protein PvdT and with the outer membrane channel OpmQ were detected in the membrane fraction and represented around 70 % of mCHERRY fluorescence signal (**Figure S4B**). Concerning the PvdR periplasmic adaptor protein, no mCHERRY was detected in the periplasmic fraction of *mcherry-pvdR* (**Figure S4B**) and most of it in the membrane fraction, as for PvdT or OpmQ. This may indicate that the association with its partners is tight enough to prevent the tripartite efflux pump dissociation during the fractionation protocol.

The growth and PVD production were monitored upon time for PAO1, PAO1*pvdRTopmQ* and our insertion mutants in iron-depleted minimal media by measuring the optical densities at 600 nm and 400 nm, respectively (**Figure S4C**). As previously reported, PVD secretion is only slightly affected in a PAO1*pvdRTopmQ* deletion strain, probably due to another efflux system taking over this process . *mcherry-pvdR*, *mcherry-pvdT* and *mcherryopmQ* strains did not present growth or PVD secretion defects (**Figure S4C**).

A phenotype associated with the PvdRTOpmQ efflux pump deletion resides in an increase in the PVD periplasmic accumulation . To investigate the functionality of our fusion proteins, we quantified the siderophore content in the periplasm. Strains were imaged by epifluorescence imaging at the spectral characteristics of PVD, keeping the same illumination for accurate comparison. When the illumination was set for appropriate imaging of PVD in PAO1*pvdRTopmQ*, the periplasmic PVD in the PAO1 strain is not detected because to low (**Figure S4D**), as previously reported . The *mcherry-opmQ* strain seemed to accumulate even more PVD in its periplasm than PAO1*pvdRtopmQ*, indicating that this fusion is somehow defective in PVD secretion (**Figure S4D**) even if PVD production is similar to wild type PAO1 (**Figure S4C**). Wild-type periplasmic PVD levels were detected in *mcherry-pvdR* and *mcherry-pvdT* strains indicating a wild-type phenotype with regards to PVD secretion (**Figure S4D**).

**
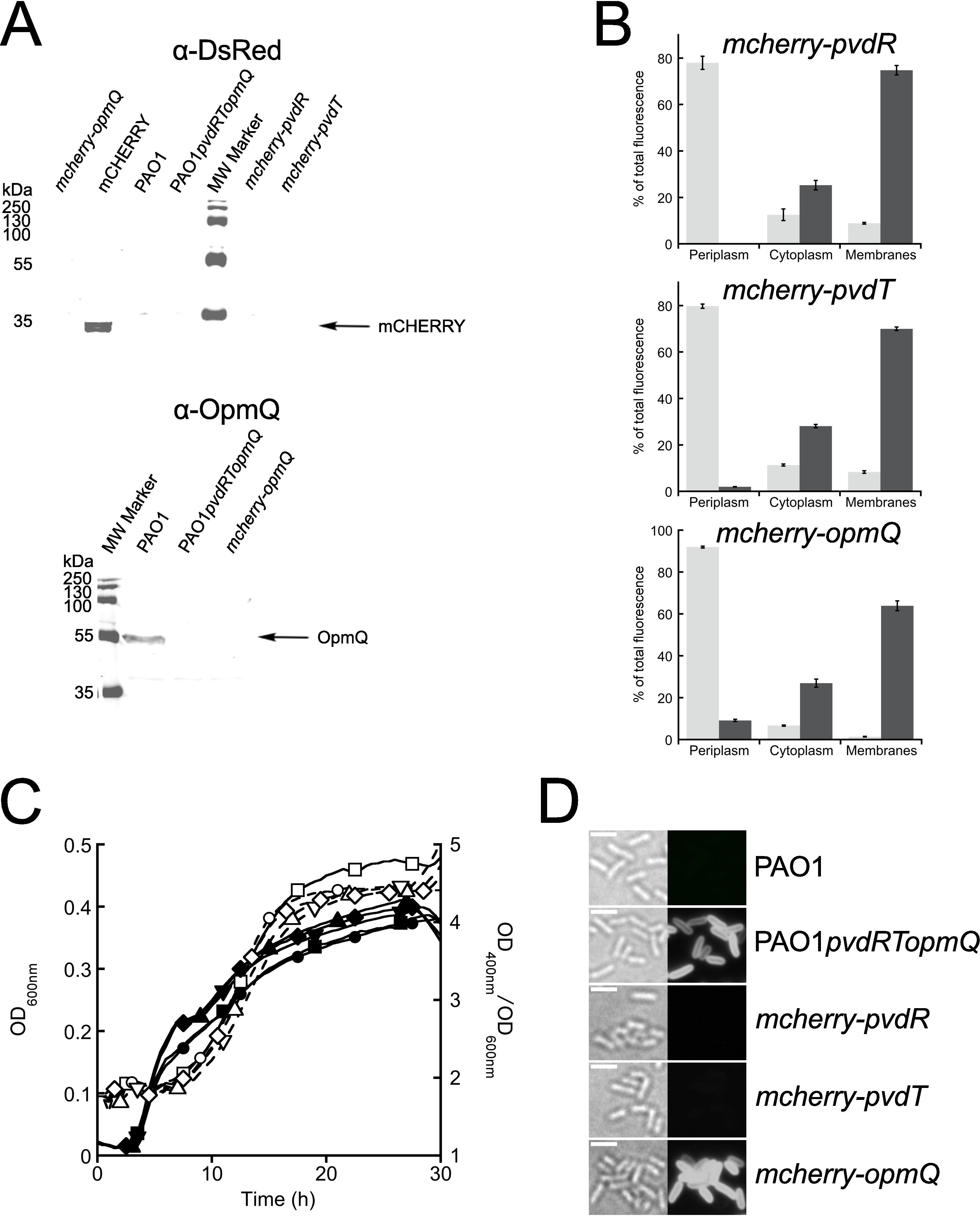
**

**Figure S4: Phenotypic characterization of *mcherry-pvdR, mcherry-pvdT*, *mcherry-opmQ* and *opmQ-mcherry*.** (**A**) Immunoblot analysis of PAO1, PAO1*pvdRTopmQ*, mCHERRY N-terminal fusion with PvdR and PvdT and OpmQ strains. The equivalent of 0.25 OD_600_ units of each strain were lysed in loading buffer, DNA digested by benzonase (1 U), loaded onto SDS PAGE for protein separation and proteins were blotted onto nitrocellulose membrane. For all strains, mCHERRY was detected using anti-DsRed antibodies (upper panel) and for *mcherry-opmQ,* PAO1 and PAO1*pvdRTopmQ*, an anti-OpmQ antibody was also used (lower panel). Molecular weight (MW) marker bands are indicated on the left. (**B**) Cellular fractionation of *mcherry-pvdR* (upper panel)*, mcherry-pvdT* (middle panel) and *mcherry-opmQ* (lower panel) strains. Fluorescence of PVD and mCHERRY were measured for equal volumes of the cytoplasmic, periplasmic and cell membranes fractions. The data are means of three independent experiments and normalized as percentages of total fluorophore fluorescence. White and dark gray columns represent Pvd and mCHERRY, respectively. (**C**) The growth (solid lines) and pyoverdine production (dashed lines, empty markers) of PAO1 (◼), PAO1*pvdRTopmQ* (⚫), *mcherry-pvdR* (▲), *mcherry-pvdT* (⯆) and *mcherry-opmQ* (◆) strains were monitored by OD_600_ and OD_400_ measurements over time. Iron-starved bacteria were inoculated in fresh minimal media and distributed in 96 wells plate. Measurements were carried out every 30 min in a Tecan microplate reader with shaking and incubation at 30°C. Every single curve is a mean over 6 replicates. PVD production (OD_400_) at a given time was normalized by corresponding OD_600_. Every single curve is a mean over 6 replicates. (**D**) PVD periplasmic content was evaluated in epifluorescence microscopy using the same illumination characteristics for all strains. Brightfield (left) and PVD fluorescence (right) images are presented (scale bar 2 µm).

**
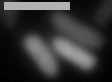
**

**Figure S5: Fluorescence microscopy analysis of fluorescent PAO1 expressing mCHERRY (strain PAO1(pMMB-*mcherry*)).** The same protocol as in the legend of Figure 1B was used (scale bar 2 µm).

**REFERENCES**

1. Voisard C, Bull C, Keel C, Laville J, Maurhofer M, et al. (1994) Biocontrol of root diseases by *Pseudomonas fluorescens* CHAO: current concepts and experimental approaches. In: O'Gara F, Dowling DN, Boesten, editors. Molecular Ecology of Rhizosphere Microorganisms. Weinheim, Germany: VCH. pp. 67-89.

2. Ye RW, Haas D, Ka JO, Krishnapillai V, Zimmermann A, et al. (1995) Anaerobic activation of the entire denitrification pathway in *Pseudomonas aeruginosa* requires Anr, an analog of Fnr. J Bacteriol 177: 3606-3609.

3. Guillon L, El Mecherki M, Altenburger S, Graumann PL, Schalk IJ (2012) High cellular organisation of pyoverdine biosynthesis in *Pseudomonas aeruginosa*: localization of PvdA at the old cell pole. Environ Microbiol 14: 1982-1994.

4. Schalk IJ, Hennard C, Dugave C, Poole K, Abdallah MA, et al. (2001) Iron-free pyoverdin binds to its outer membrane receptor FpvA in *Pseudomonas aeruginosa*: a new mechanism for membrane iron transport. Mol Microbiol 39: 351-360.

5. Clément E, Mesini PJ, Pattus F, Abdallah MA, Schalk IJ (2004) The binding mechanism of pyoverdin with the outer membrane receptor FpvA in *Pseudomonas aeruginosa* is dependent on its iron-loaded status. Biochemistry 43: 7954-7965.

6. Voisard C, Bull CT, Keel C, Laville J, Maurhofer M, et al. (1994) Biocontrol of root diseases by *Pseudomonas fluorescens* CHA0: current concepts and experimental approaches. Molecular Ecology of Rhizosphere Microorganisms (O'Gara, F, Dowling, DN, and Boesten, Eds): VCH, Weinheim, Germany. pp. 67-89.

7. Morales VM, Backman A, Bagdasarian M (1991) A series of wide-host-range low-copy-number vectors that allow direct screening for recombinants. Gene 97: 39-47.

8. Tiburzi F, Imperi F, Visca P (2008) Intracellular levels and activity of PvdS, the major iron starvation sigma factor of *Pseudomonas aeruginosa*. Mol Microbiol 67: 213-227.

9. Yeterian E, Martin LW, Guillon L, Journet L, Lamont IL, et al. (2010) Synthesis of the siderophore pyoverdine in *Pseudomonas aeruginosa* involves a periplasmic maturation. Amino Acids 38: 1447-1459.

10. Brillet K, Ruffenach F, Adams H, Journet L, Gasser V, et al. (2012) An ABC transporter with two periplasmic binding proteins involved in iron acquisition in *Pseudomonas aeruginosa*. ACS Chem Biol 7(12):2036-45.

11. Chen JC, Viollier PH, Shapiro L (2005) A membrane metalloprotease participates in the sequential degradation of a Caulobacter polarity determinant. Mol Microbiol 55: 1085-1103.

12. Postle K, Larsen RA (2007) TonB-dependent energy transduction between outer and cytoplasmic membranes. Biometals 20: 453-465.

13. Hannauer M, Yeterian E, Martin LW, Lamont IL, Schalk IJ (2010) Secretion of newly synthesized pyoverdine by *Pseudomonas aeruginosa* involves an efflux pump. FEBS Lett 584: 4751-4755.

14. Imperi F, Tiburzi F, Visca P (2009) Molecular basis of pyoverdine siderophore recycling in *Pseudomonas aeruginosa*. Proc Natl Acad Sci U S A 106: 20440-20445.

15. Yeterian E, Martin LW, Lamont IL, Schalk IJ (2010) An efflux pump is required for siderophore recycling by *Pseudomonas aeruginosa*. Environ Microbiol Report 2: 412-418.

16. Hannauer M, Yeterian E, Martin LW, Lamont IL, Schalk IJ (2010) An efflux pump is involved in secretion of newly synthesized siderophore by *Pseudomonas aeruginosa*. FEBS Lett 584: 4751-4755.
